# Supplementary material for: zGrad is a nanobody-based degron system that inactivates proteins in zebrafish
Source: eLife. 2019 Feb 8;8:e43125. doi: 10.7554/eLife.43125 (PMC6384026; doi:10.7554/eLife.43125)
Supplement: Transparent reporting form [file elife-43125-transrepform.docx]

***eLife’s* transparent reporting form**

We encourage authors to provide detailed information *within their submission* to facilitate the interpretation and replication of experiments. Authors can upload supporting documentation to indicate the use of appropriate reporting guidelines for health-related research (see [EQUATOR Network](http://www.equator-network.org/%20)), life science research (see the [BioSharing Information Resource](https://biosharing.org/)), or the [ARRIVE guidelines](http://www.plosbiology.org/article/info:doi/10.1371/journal.pbio.1000412) for reporting work involving animal research. Where applicable, authors should refer to any relevant reporting standards documents in this form.

If you have any questions, please consult our Journal Policies and/or contact us: [editorial@elifesciences.org](mailto:editorial@elifesciences.org).

**Sample-size estimation**

- You should state whether an appropriate sample size was computed when the study was being designed
- You should state the statistical method of sample size computation and any required assumptions
- If no explicit power analysis was used, you should describe how you decided what sample (replicate) size (number) to use

Please outline where this information can be found within the submission (e.g., sections or figure legends), or explain why this information doesn’t apply to your submission:

We did not estimate the sample size a priori. In many of the reported experiments, each cross of fish yields only a small number of embryos of the desired genotype so we aimed for at least three replicates with a sample size of at least 10 embryos.

Based on the sample size (often pooled from replicates) we determined the p values. The information about the number of replicates, sample size and p values are in the figures, the figure legends and the material and methods section.

**Replicates**

- You should report how often each experiment was performed
- You should include a definition of biological versus technical replication
- The data obtained should be provided and sufficient information should be provided to indicate the number of independent biological and/or technical replicates
- If you encountered any outliers, you should describe how these were handled
- Criteria for exclusion/inclusion of data should be clearly stated
- High-throughput sequence data should be uploaded before submission, with a private link for reviewers provided (these are available from both GEO and ArrayExpress)

Please outline where this information can be found within the submission (e.g., sections or figure legends), or explain why this information doesn’t apply to your submission:

In each panel, we provide the information of biological replicates as N. In some instances, we decided to compare only the samples (control samples and experimental samples) from one replicate because some experimental parameters are difficult to keep the same. This applies to the heat shock experiments where the exact timing of the heat shock in the water bath cannot be perfectly replicated. In the cases, where we pooled the samples from different replicates we state so in the methods section.

Our mRNA injection experiments sometimes result in embryos that have not been injected or have been injected with very little volume/mRNA. Such embryos were removed from the dataset based on low or no GFP expression and defined as outliers.

**Statistical reporting**

- Statistical analysis methods should be described and justified
- Raw data should be presented in figures whenever informative to do so (typically when N per group is less than 10)
- For each experiment, you should identify the statistical tests used, exact values of N, definitions of center, methods of multiple test correction, and dispersion and precision measures (e.g., mean, median, SD, SEM, confidence intervals; and, for the major substantive results, a measure of effect size (e.g., Pearson's r, Cohen's d)
- Report exact p-values wherever possible alongside the summary statistics and 95% confidence intervals. These should be reported for all key questions and not only when the p-value is less than 0.05.

Please outline where this information can be found within the submission (e.g., sections or figure legends), or explain why this information doesn’t apply to your submission:

We included the statements for statistical tests we employed in the material and method section as “Statistical analysis.” We included exact value of N by the beeswarm plots, definition of center (mean), dispersion (S.D.) in the each panel in figures and figure legends. We included the definition of statistical significance, such as p<0.01 or N.S. = p>0.05 in the figure legends.

(For large datasets, or papers with a very large number of statistical tests, you may upload a single table file with tests, Ns, etc., with reference to sections in the manuscript.)

**Group allocation**

- Indicate how samples were allocated into experimental groups (in the case of clinical studies, please specify allocation to treatment method); if randomization was used, please also state if restricted randomization was applied
- Indicate if masking was used during group allocation, data collection and/or data analysis

Please outline where this information can be found within the submission (e.g., sections or figure legends), or explain why this information doesn’t apply to your submission:

This section is not applicable for our study.

**Additional data files (“source data”)**

- We encourage you to upload relevant additional data files, such as numerical data that are represented as a graph in a figure, or as a summary table
- Where provided, these should be in the most useful format, and they can be uploaded as “Source data” files linked to a main figure or table
- Include model definition files including the full list of parameters used
- Include code used for data analysis (e.g., R, MatLab)
- Avoid stating that data files are “available upon request”

Please indicate the figures or tables for which source data files have been provided:

We provided two tables: Table 1 as a detail numerical data set for supporting Cdh1 transgene is fully functional. Table 2 as detail numerical data set for protein degradation kinetics exported from fitting against the one-exponential decay model.

We provided three Data Files (Data Files 1-3), which are files describing the custom Image J macros we have written to analyze our data. We provided these files in the .txt format.
